# Supplementary material for: PneumoniaCheck, a novel aerosol collection device, permits capture of airborne Mycobacterium tuberculosis and characterisation of the cough aeromicrobiome in people with tuberculosis
Source: Ann Clin Microbiol Antimicrob. 2024 Aug 22;23:74. doi: 10.1186/s12941-024-00735-x (PMC11342687; doi:10.1186/s12941-024-00735-x)
Supplement: Supplementary file 2 — Supplementary Material 2 [file 12941_2024_735_MOESM2_ESM.docx]

**Contents**

**Methods**…………………………………………………………………………………………..3

*Oral wash and bronchoalveolar lavage (BALF) sample collection and processing……….*3

**Supplementary figures**…………………………………………………………………………3

*Figure S1. Sequencing read counts*…………………………………………………………….3

*Figure S2. Background controls*………………………………………………………………….4

*Figure S3. Identification of background contaminating genera*……………………………….5

*Figure S4. Bray-Curtis distances………………………………………………………………... 6*

*Figure S5. Bronchoalveolar lavage fluid (BALF) is Mycobacterium-enriched relative to sputum and oral washes…*………………………………………………………………………. 7

**References**…………………………………………………………………………………………8

# **Methods**

## *Oral wash and bronchoalveolar lavage (BALF) sample collection and processing*

People provided an oral wash sample by gargling ~20mL of sterile water for ~10s. Bronchoalveolar lavage fluid (BALF) was collected during a bronchoscopy with bronchoalveolar lavage performed according to the European Respiratory Society (ERS) and American Thoracic Society (ATS) recommendations^1,2^. In brief after sedation and pre-oxygenation the bronchoscope was advanced (without suctioning until wedged in the target site) to the site of disease and bronchoalveolar lavage was performed in 50mL increments up to a total volume of 100mL.

# **Supplementary figures**

##
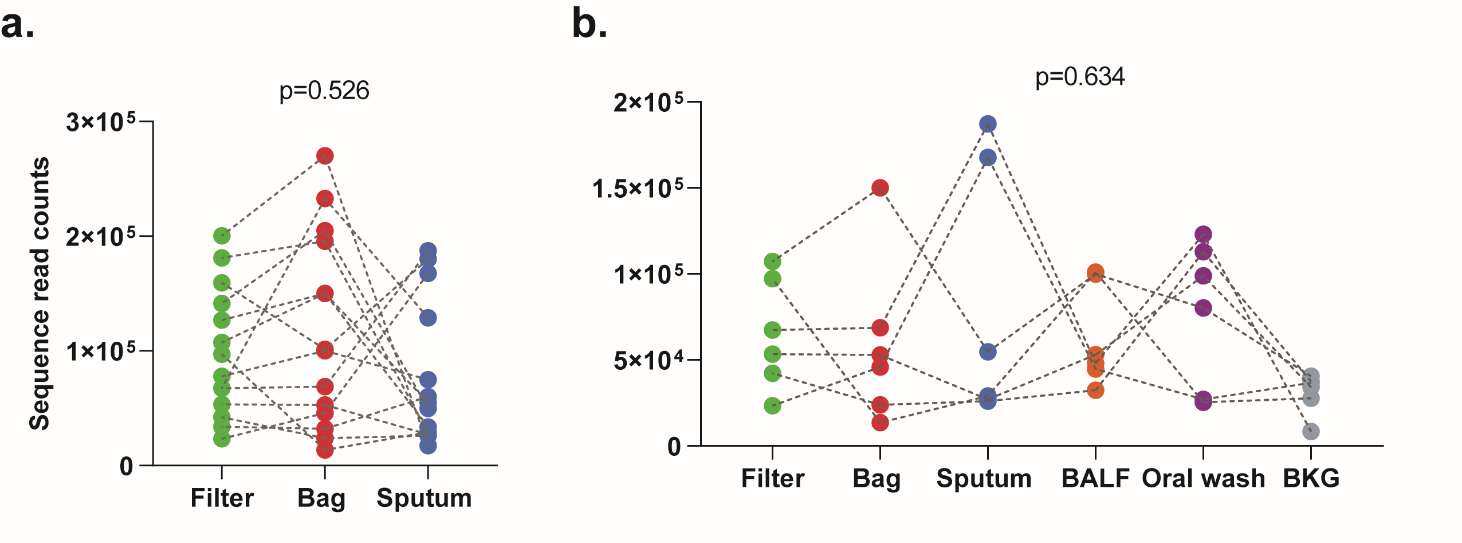
**Figure S1. Sequencing read counts:** Paired sequence read count comparison between **(a)** aerosols and sputum **(b)** aerosols, sputum, bronchoalveolar lavage (BALF), oral wash and DNA background sampling controls (BKG).

##
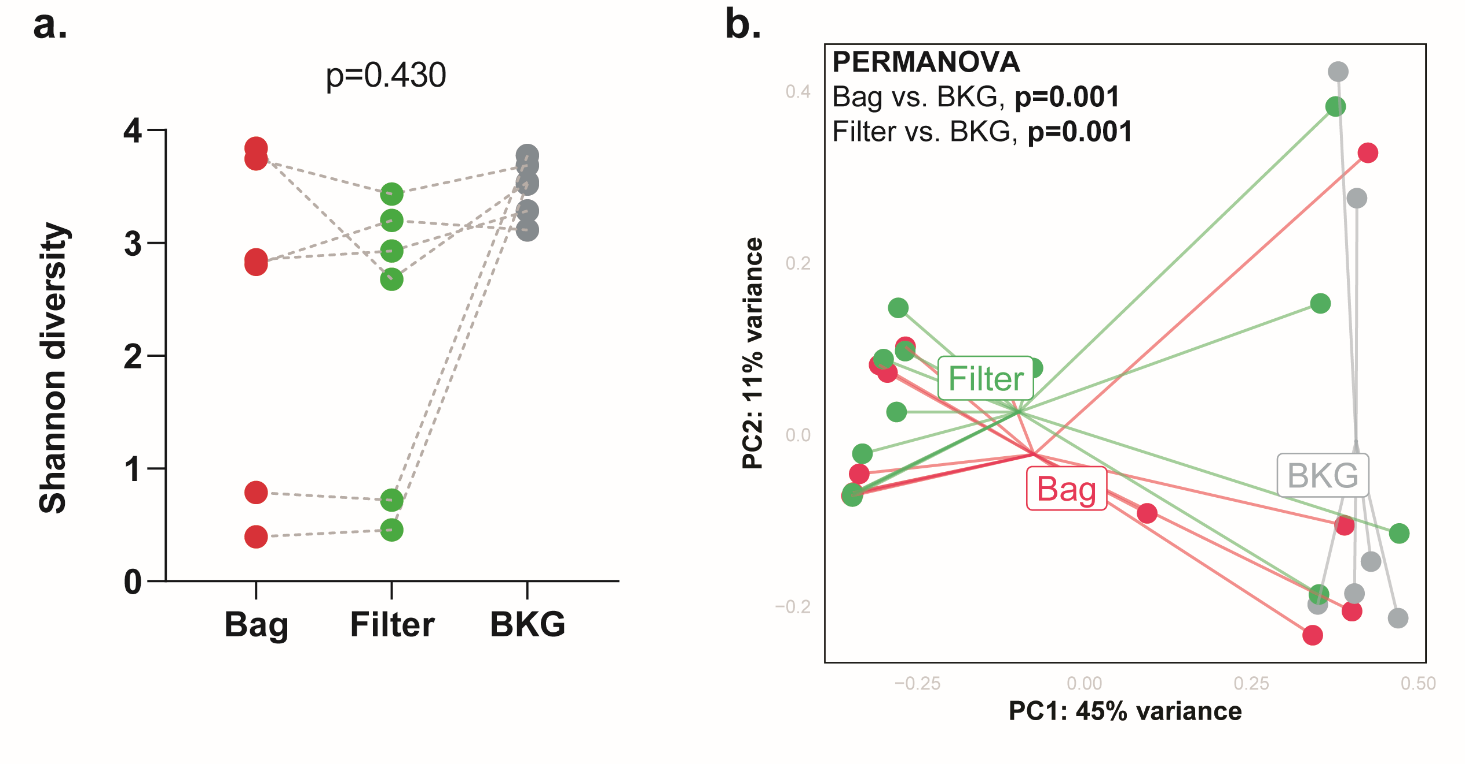
**Figure S2. Background controls:** **(a)** Paired α-diversity comparison between aerosols and DNA background sampling controls (BKG) showing similarity. **(b)** ꞵ-diversity analysis showing distinct microbial composition between aerosols and BKG. It is unlikely aerosol microbial composition is a result of contamination.

##
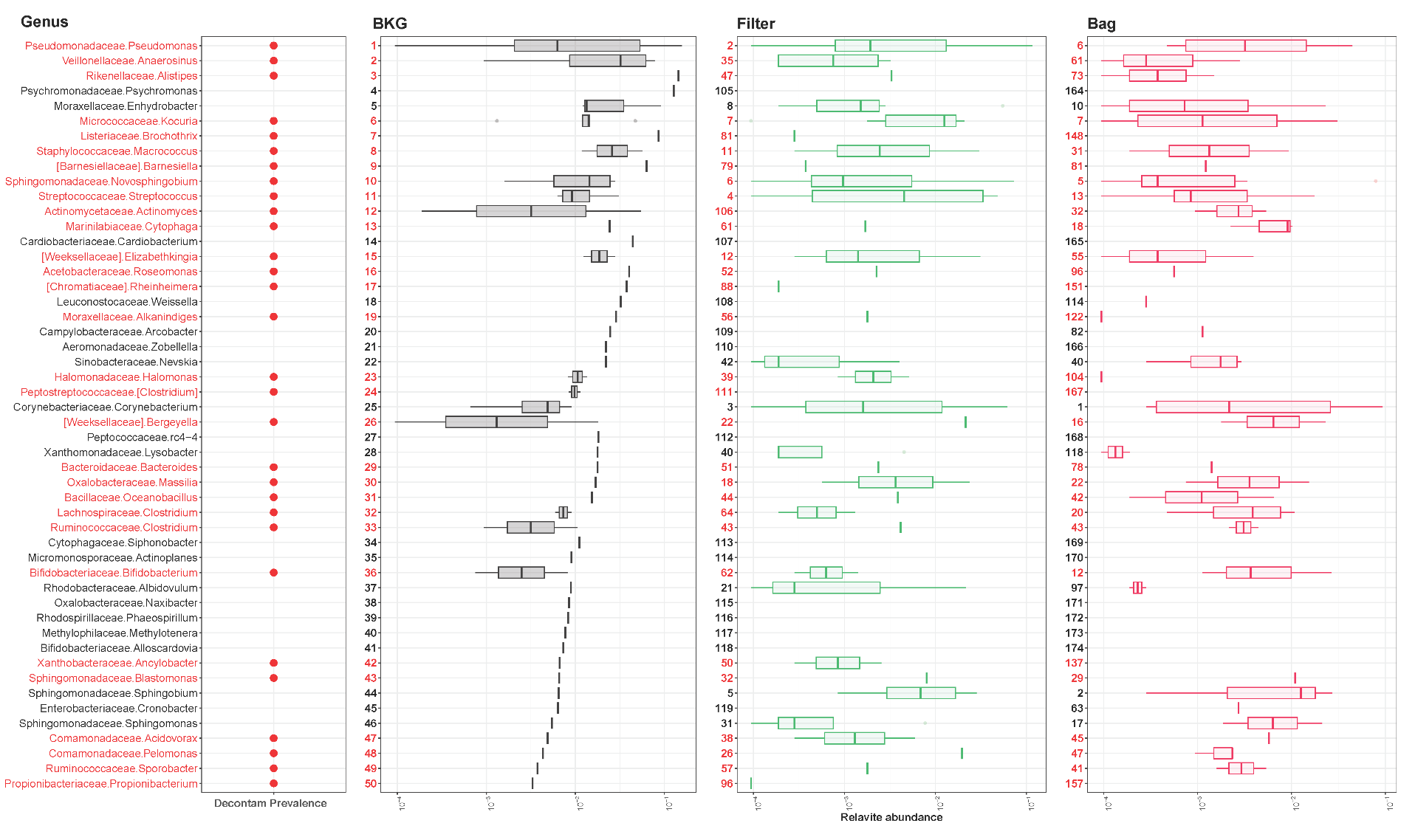
**Figure S3. Identification of background contaminating genera:** List of top 50 potentially contaminating taxa ranked by most-to-least relative abundance in DNA background sampling controls (BKG). Red indicates taxa identified are bioinformatically identified as potential contaminants.


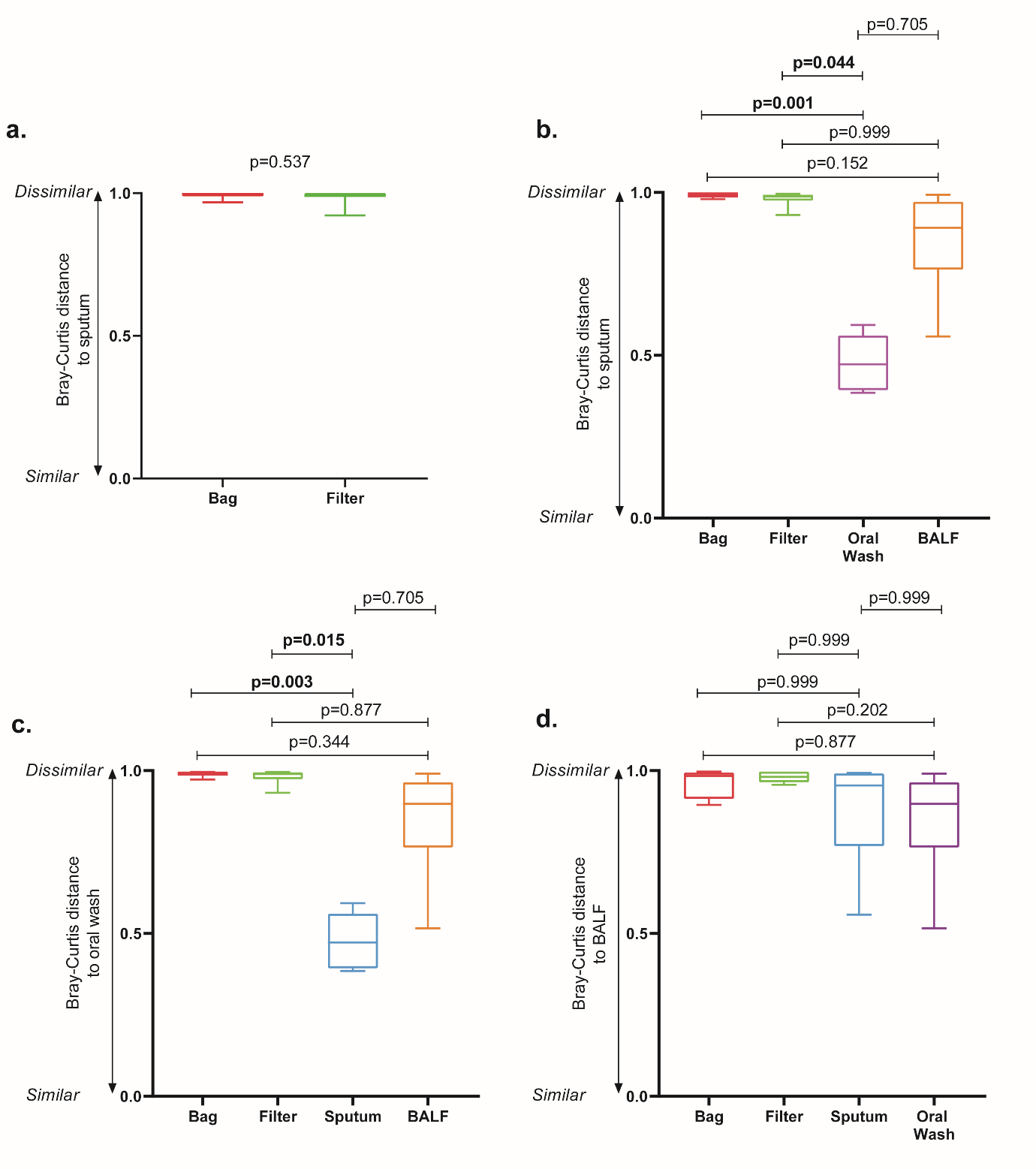
**Figure S4.** **Bray-Curtis distances**: Illustrating microbial composition dissimilarity (similar samples have distances closer to zero) of (a) aerosols to sputum (b) aerosols, oral wash and bronchoalveolar lavage fluid (BALF) to sputum (c) aerosols, sputum and BALF to oral wash and (d) aerosols, sputum, and oral wash to BALF.

**Figure S5.** **Bronchoalveolar lavage fluid (BALF) is Mycobacterium-enriched relative to sputum and oral washes.** Volcano plots showing differentially abundant taxa in (a) sputum vs. BALF, and (b) oral wash vs. BALF. Taxa that are considered discriminatory appear above threshold (marked by the red dotted line, FDR=0.2). The size of the dots corresponds to the relative abundance of the taxa.


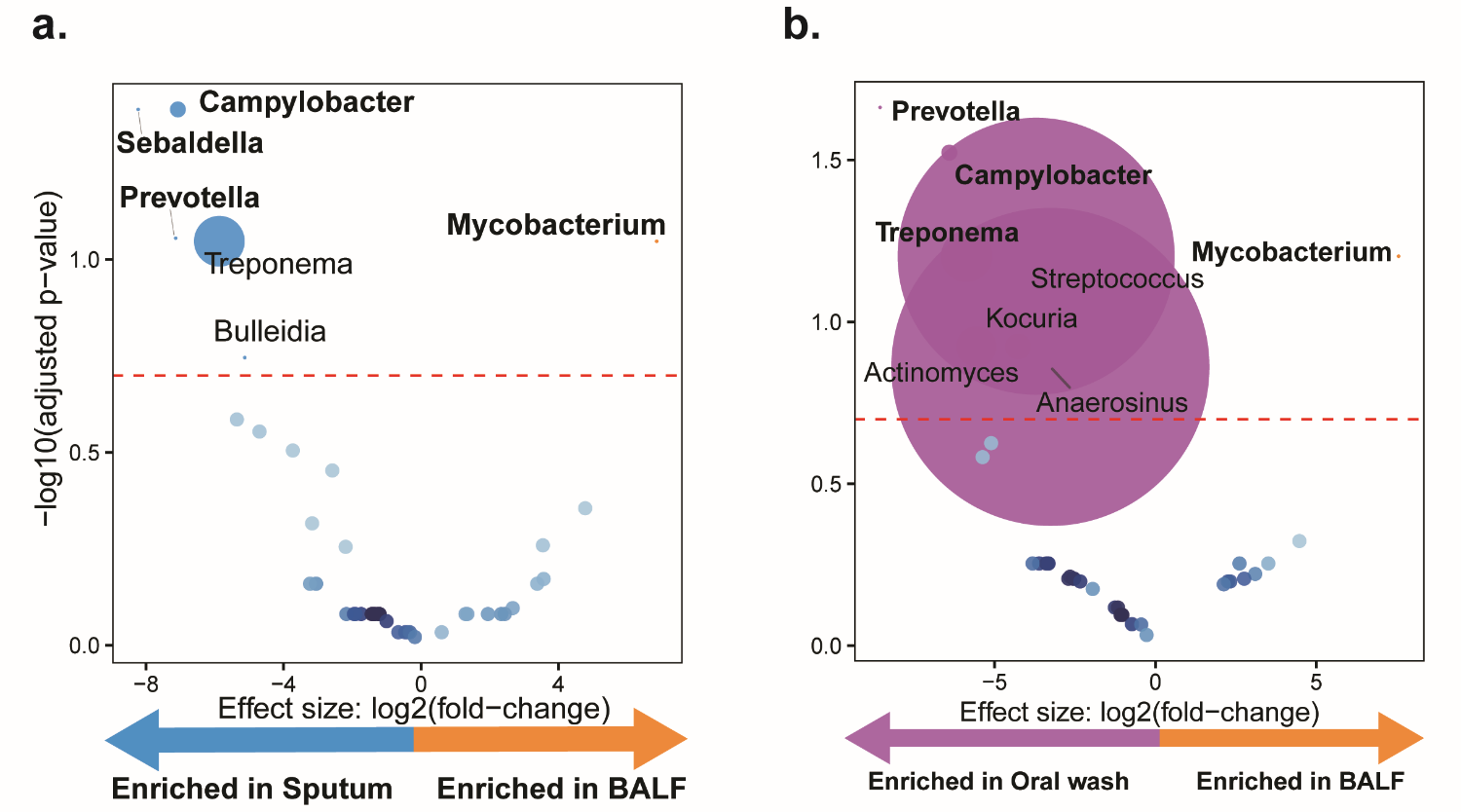


# **References**

1. Haslam PL, Baughman RP. Report of ERS Task Force: guidelines for measurement of acellular components and standardization of BAL. *European Respiratory Journal* 1999; **14**(2): 245-8.

2. Meyer KC, Raghu G, Baughman RP*, et al.* An official American Thoracic Society clinical practice guideline: the clinical utility of bronchoalveolar lavage cellular analysis in interstitial lung disease. *American journal of respiratory and critical care medicine* 2012; **185**(9): 1004-14.
